# A Novel Nomogram and Risk Classification System Predicting the Cancer-Specific Survival of Muscle-Invasive Bladder Cancer Patients after Partial Cystectomy

**DOI:** 10.1155/2022/2665711

**Published:** 2022-03-01

**Authors:** Xiangpeng Zhan, Tao Chen, Ming Jiang, Wen Deng, Xiaoqiang Liu, Luyao Chen, Bin Fu

**Affiliations:** ^1^Department of Urology, The First Affiliated Hospital of Nanchang University, Nanchang, Jiangxi, China; ^2^Department of Health Statistics, Second Military Medical University, Shanghai, China

## Abstract

**Purpose:**

To establish a prognostic model that estimates cancer-specific survival (CSS) probability for muscle-invasive bladder cancer patients undergoing partial cystectomy. *Patients and Methods*. 866 patients from the Surveillance, Epidemiology, and End Results (SEER) database (2004–2015) were enrolled in our study. These patients were randomly divided into the development cohort (*n* = 608) and validation cohort (*n* = 258) at a ratio of 7 : 3. A Cox regression was performed to select the predictors associated with CSS. The Kaplan–Meier method was used to analyze the survival outcome between different risk groups. The calibration curves, receiver operating characteristic (ROC) curves, and the concordance index (C-index) were utilized to evaluate the performance of the model.

**Results:**

The nomogram incorporated age, histology, T stage, N stage, M stage, regional nodes examined, and tumour size. The C-index of the model was 0.733 (0.696–0.77) in the development cohort, while this value was 0.707 (0.705–0.709) in the validation cohort. The AUC of the nomogram was 0.802 for 1-year, 0.769 for 3-year, and 0.799 for 5-year, respectively, in the development cohort, and was 0.731 for 1-year, 0.748 for 3-year, and 0.752 for 5-year, respectively, in the validation cohort. The calibration curves for 1-year, 3-year, and 5-year CSS showed great concordance. Significant differences were observed between high, medium, and low risk groups (*P* < 0.001).

**Conclusions:**

We have constructed a highly discriminative and precise nomogram and a corresponding risk classification system to predict the cancer-specific survival for muscle-invasive bladder cancer patients undergoing partial cystectomy. The model can assist in the decision on choice of treatment, patient counselling, and follow-up scheduling.

## 1. Introduction

Bladder cancer (BC) is the most common cancer of the urinary system, and it is the cause of death of 3% of patients with cancer in the UK [1, 2]. Approximately 25% of patients newly diagnosed with bladder cancer present with muscle-invasive bladder cancer (MIBC). MIBC is a heterogeneous and serious disease with a high recurrence and mortality rate [3]. A retrospective study demonstrated that 86% and 48% of bladder cancer-specific mortality were observed between MIBC patients receiving treatment and those without, respectively [4].

It is a considerable challenge for the urologist to choose suitable treatments for MIBC patients considering their worse prognosis. Radical cystectomy (RC) with bilateral pelvic lymph node dissection (PLND) is still the gold-standard treatment for MIBC patients nowadays [3].However, accompanying potential complications such as severe blood loss, infection, and paralytic intestinal obstruction, as well as an unsatisfactory patient's quality of life made MIBC patients unwilling to accept this approach [5, 6]. Urologists are committed to exploring bladder preservation strategies that can balance patients' quality of life and the oncological outcome and provide an alternative for those unwilling or unfit to undergo radical cystectomy.

Partial cystectomy (PC) as a method of bladder preservation has been proposed as an alternative treatment for radical cystectomy [7].Over the past decade, a growing body of studies indicated that partial cystectomy presented a comparable outcome with radical cystectomy. A retrospective study based on the SEER database showed that partial cystectomy could obtain similar overall survival (OS) and cancer-specific survival (CSS) compared to RC in appropriately selected patients [8]. Similarly, a study enrolling 58 patients undergoing PC observed acceptable outcomes with 74% overall survival and 67% disease-free survival in highly selected patients with MIBC [9]. All these results seemed to confirm that PC was a worth trying bladder preservation method for appropriately selected MIBC patients. Therefore, developing the appropriate criteria which could pick out MIBC patients with a better prognosis was the most significant step.

A nomogram is a visible and trustworthy statistical prediction tool widely used to provide tailored individual prognostic information. The nomogram incorporated several essential factors associating with the prognostic endpoint such as significant demographic, clinical, pathological, and treatment features. Individual survival probability and risk stratification could be obtained from the prognostic nomogram [10]. MD Anderson has developed strict criteria for MIBC patients undergoing PC, including a solitary tumour allowing for 2 cm margins, no carcinoma in situ (CIS), and no history of bladder cancer [11]. However, the insufficient number of patients and the lack of a statistical prediction tool made the criteria unsatisfactory. Thus, it is imperative to construct a prognostic nomogram for MIBC patients undergoing PC.

In our study, we searched MIBC patients undergoing PC from the Surveillance, Epidemiology, and End Results (SEER) database from 2004 to 2015 to analyze significant factors associated with CSS of MIBC patients undergoing PC. We were committed to constructing a prognostic nomogram to predict CSS in MIBC patients undergoing PC and suggest treatment for these patients. In addition, we evaluated the performance and internally verified the applicability of the nomogram to make the predictive model more convincing.

## 2. Patients and Method

### 2.1. Data Source and Population Selection

The patient data comes from the Surveillance, Epidemiology, and End Results (SEER) (2004–2015) database, which well demonstrates the patient demographic and cancer information of the US population. The case listing was searched from the dataset of Incidence - SEER Research Plus Data, 18 Registries, Nov 2019 Sub (2000–2017). Meanwhile, SEER*∗*Stat version 8.3.9 was used to select patients and collect data. The inclusion criteria were as follows: (a) diagnosed from 2004 to 2015; (b) AJCC T stage: T2, T3, and T4; (c) surgical approach: partial cystectomy (coded 30); and (d) histology: transitional cell carcinoma (TCC), squamous cell carcinoma (SCC), and adenocarcinoma. The exclusion criteria in this study were as follows: (1) race unknown (*n* = 2); (2) tumor grade unknown (*n* = 122); (3) Nx (*n* = 49); (4) Mx (*n* = 14); (5) marital status unknown (*n* = 48); unknown survival time (*n* = 16); and (6) other types of histology (*n* = 199).

### 2.2. Definition of Variables and Endpoint

The variables from the SEER database included four parts such as demographic characteristics (such as age at diagnosis, gender, race, and marital status), tumour characteristics (such as tumour grade, histology, TNM stage, tumour size, and number of tumours), treatment information (such as radiotherapy, chemotherapy, and regional nodes examined), and survival information (survival month and SEER cause-specific death classification).

Based on the “RX Summ-Surg Prim Site (1998+)” column in the SEER database, partial cystectomy was coded as 30. The continuous variables including age, tumour size, and the number of tumours and regional nodes examined were transformed into categorical variables: age (<60, 60–70, 70–80, and >80); tumour size (<3 cm and ≥3 cm); number of tumours (single and multiple); and positive lymph nodes (1, 1–3, and >3). Other variables included the following: (1) race (white, black, and others including American Indian, Alaska Native, Asian, and Pacific Islander); (2) sex (male and female); (3) marital status (married; single; separated, divorced or widowed (SDW)); (4) grade (Grade I or Grade II; Grade III or Grade IV); (5) histology (transitional cell carcinoma (TCC); squamous cell carcinoma (SCC)); (6) T stage (T2, T3, and T4); (7) N stage (N0 and N1–N3); (8) M stage (M0 and M1); (9) radiotherapy (no/unknown and yes); and (10) chemotherapy (no/unknown and yes).

Cancer-specific death was defined as the underlying death cause of bladder cancer according to the “SEER cause-specific death classification” column in the SEER database. The primary endpoint in this study was cancer-specific survival (CSS), which was the period from the initial diagnosis of bladder cancer to BC-specific death.

### 2.3. Statistical Analysis

All patients available were randomly split into the development and validation cohorts at the rate of 7 : 3, and the development cohort was applied to establish the predictive nomogram and the risk classification system. Internal verification was performed in the validation cohort.

The univariate Cox regression analysis was the first step to select variables in the development cohort, and those variables whose *P*value of 0.05 or less would be included in the multivariate analysis. The backwards model selection procedure was used in the multivariate analysis. All results in the Cox regression analysis were shown as hazards ratios (HR) and 95% confidence intervals (95% CI). Finally, the factors associated with CSS were utilized to construct the nomogram and risk classification system.

A nomogram was constructed for visualized prediction of 1-, 3-, and 5-year survival probabilities in the development cohort. The performance of the prediction model was evaluated by the concordance index (C-index), the receiver operating characteristic (ROC) curves with the calculated area under the curve (AUC), and calibration curves. The C-index and AUC were used to access the nomogram's predictive accuracy and discrimination ability, while the calibration curves (100 bootstrap resamples) were utilized to compare the concordance of predicted and actual outcomes of 1-, 3-, and 5-year survival times. Meanwhile, we used the Kaplan–Meier method with the log-rank test to analyze the survival outcome between three risk groups.

SPSS 22.0 (IBM Corp, Armonk, NY) and R version 3.6.3 were used for all statistical analyses. All the results' *P*values were two-tailed, and a *P* < 0.050 was considered significant.

## 3. Results

### 3.1. Patient Characteristics

Finally, 866 patients were enrolled in our study, and there were 608 patients in the development cohort and 258 patients in the validation cohort. As shown in Table 1, no statistical differences in demographic information and clinical characteristics were observed between the development and validation cohorts. The median follow-up time was 35.8 months for all patients. The mean survival time in the development cohort was 48.63 months, while it was 55.78 months in the validation cohort (Table 1). At the end of follow-up, 395 (45.6%) patients died of bladder cancer in all patients, and there were 291 (47.9%) patients in the development cohort and 104 (40.3%) patients in the validation cohort. The 1-, 3-, and 5-year CSS rates were 75.32%, 50.65%, and 31.9%, respectively, in the development cohort. Meanwhile, the 1-, 3-, and 5-year CSS rates were 75.19%, 54.26%, and 38.37%, respectively, in the validation cohort.

### 3.2. Identification of Predictive Factors

The Cox proportional hazards model was used to evaluate the prediction power of factors. The results of the multivariate analysis were as following: age 70–80 vs. age <60 (HR = 1.633, 95% CI: 1.075–2.479, *P* = 0.021); age >80 vs. age <60 (HR = 2.289, 95% CI: 1.525–3.434, *P* < 0.001); squamous cell carcinoma vs. transitional cell carcinoma (HR = 2.447, 95% CI: 1.537–3.894, *P* < 0.001); T3 vs. T2 (HR = 3.395, 95% CI: 2.508–4.597, *P* < 0.001) and T4 vs. T2 (HR = 3.956, 95% CI: 2.668–5.866, *P* < 0.001); M1 vs. M0 (HR = 4.872, 95% CI: 2.965–8.007, *P* < 0.001); tumor size >3 cm vs. tumor size <3 cm (HR = 1.334, 95% CI: 2.668–5.866, *P* < 0.001); and regional nodes examined 1–3 vs. non (HR = 0.568, 95% CI: 0.396–0.815, *P* = 0.002) and regional nodes examined >3 vs. non (HR = 0.538, 95% CI: 0.406–0.713, *P* < 0.001) (Table 2). Ultimately, seven factors including age, T stage, N stage, M stage, histology, tumor size, and regional nodes examined, were identified as independent predictors of CSS for the final predictive model.

### 3.3. Building and Validating the Nomogram for CSS

**Figure 1:**
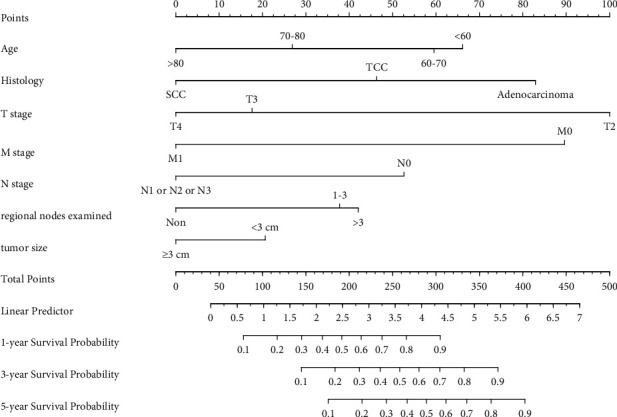
Nomogram predicting 1-, 3-, and 5-year bladder cancer-specific survival probabilities for muscle-invasive bladder cancer patients undergoing partial cystectomy. The variables include age, histology, T stage, N stage, M stage, regional nodes examined, and tumour size. Its use is to locate patient values at each axis. Draw a vertical line to the “Point” axis to determine how many points are attributed for each variable value. Sum the points for all variables. Locate the sum on the “Total Points” line. Draw a vertical line towards the 1 Yrs.Surv. Prob, 3Yrs.Surv. Prob., and 5Yrs.Surv. Prob, and Prob. axes to determine the 1-, 3-, and 5-year survival probabilities.

**Figure 2:**
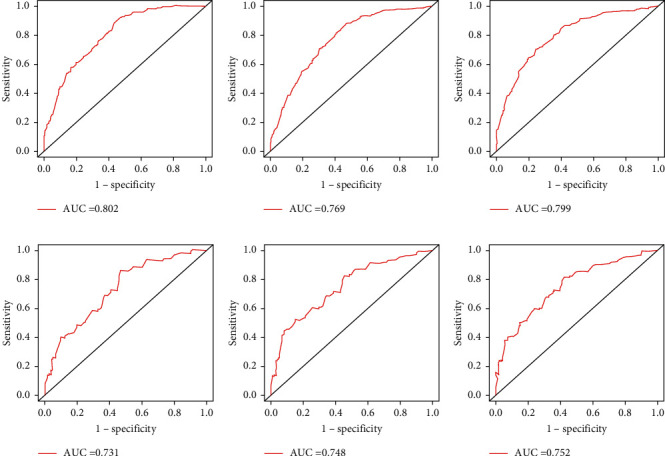
ROC curves of the nomogram predicting CSS for 1-year (a), 3-year (b), and 5-year (c) in the development cohort; ROC curves of the nomogram predicting CSS for 1-year (d), 3-year (e), and 5-year (f) in the validation cohort.

**Figure 3:**
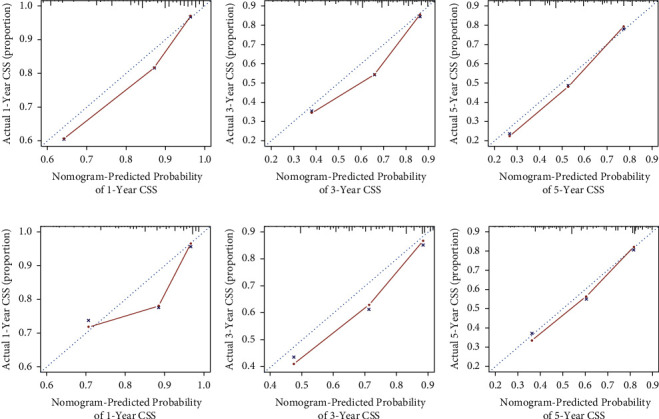
Calibration plots of the nomogram for 1-year (a), 3-year (b), and 5-year (c) in the development cohort; calibration plots of the nomogram for 1-year (d), 3-year (e), and 5-year (f) in the validation cohort.

A model predicting the 1-, 3-, and 5-year CSS of MIBC patients was virtually presented in the form of a nomogram and was validated in the validation cohort (Figure 1). The C-index of this nomogram for CSS was 0.733 (0.696–0.77) in the development cohort, while this value was 0.707 (0.705–0.709) in the validation cohort. These results were all higher than 0.666 of the TNM system. Meanwhile, the ROC curve was applied to access the discriminative ability of the nomogram, and the AUC of the model was significantly higher than the TMN system for 1-year (0.802 vs. 0.697), 3-year (0.769 vs. 0.7), and 5-year (0.799 vs. 0.736) CSS prediction in the development cohort (Figures 2(a)–2(c)). Meanwhile, the AUC of the model in the validation cohort were all higher than the TNM system (0.731 vs. 0.697 for 1-year, 0.748 vs. 0.703 for 3-year, and 0.752 vs. 0.707 for 5-year, respectively) (Figures 2(d)–2(f)). In addition, the calibration curves also showed good consistency in the probability of 1-, 3-, and 5-year CSS between actual observations and predicted outcomes both in the development and validation cohorts (Figure 3). All these results demonstrated the good performance and application of our predicted model.

### 3.4. Risk Classification System

**Figure 4:**
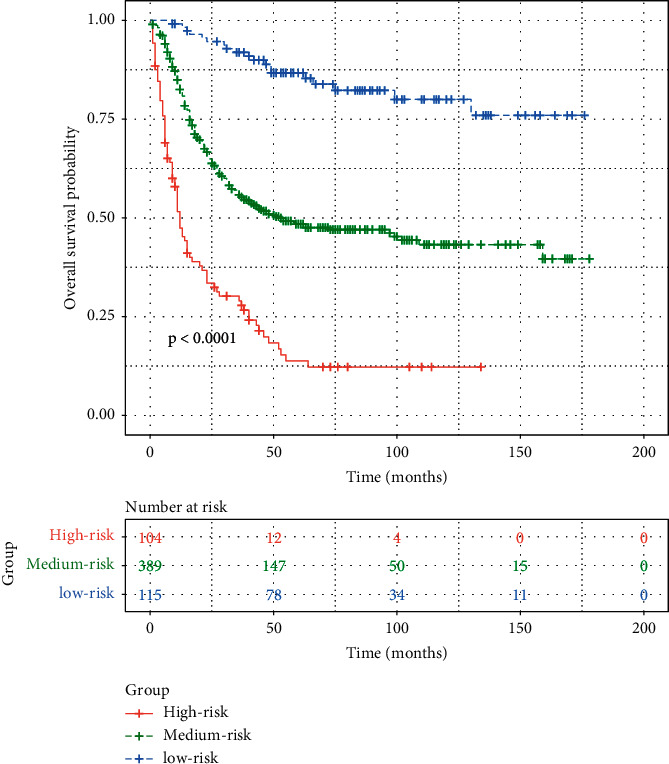
Survival curves stratified by the score calculated by the nomogram in muscle-invasive bladder cancer patients undergoing partial cystectomy. Low-risk group (score>198); medium group (score 148–198); high-risk group (score <148).

According to the contribution to this prediction model, all factors in the nomogram were granted a corresponding score between 0 and 100 (Table 3). A risk classification system for CSS in NMIBC patients undergoing PC was established based on the total scores obtained by the nomogram. Based on the new classification system, all patients in the development cohort were divided into three subgroups: low-risk group (115/608, score >280); medium group (389/608, score 190–280); and high-risk group (104/608, score<190). According to survival curves in the development cohort, significant differences were observed between low-risk, medium-risk, and high-risk groups (Figure 4).

## 4. Discussion

Muscle-invasive bladder cancer (MIBC) is usually considered as an advanced regional stage ranging from the T2 stage (which invades the muscularis propria) to the T4 stage (which invades the prostate, uterus, vagina, bowel or abdominal wall) [3]. In most guidelines, radical cystectomy is still defined as the gold-standard treatment for MIBC patients relying on better regional tumour control [3, 12]. However, it might be over-treatment for some MIBC patients who were at a relatively early stage like the T2a stage (which invades the inner half of the muscular propria) or just with a single tumour but without metastasis. Partial cystectomy as a treatment with fewer complications and no bad oncological effects has been proposed as a bladder preservation method for MIBC patients [7, 13]. Compared with RC, partial cystectomy was represented as a less morbid operation that could maintain a complete bladder and relatively acceptable voiding and sexual function. Meanwhile, a single-center retrospective study enrolling 37 MIBC patients obtained the results: the 5-year OS, CSS, and recurrence-free survival (RFS) rates were 67%, 87%, and 39%, respectively [11]. In addition, no statistical differences in OS and CSS were observed between patients undergoing RC and PC [8]. PC seemed to obtain acceptable survival outcomes when compared to RC in highly selected patients. Therefore, a scientific approach to select suitable MIBC patients who can benefit from PC was critical. The nomogram, which could precisely and scientifically provide individual survival probability at a particular time, was a suitable prediction tool to empower patients and urologists to make informed decisions.

In this study, a highly accurate and discriminating nomogram to predict cancer-specific survival of MIBC patients undergoing PC was constructed and internally validated. A univariate and multivariate analysis was performed to identify the meaningful variables associated with CSS to yield a highly accurate and streamlined prediction model. The model based on the interaction of the selected variables produced a user-friendly interface to evaluate the risk of MIBC patients after PC, and it also obtained far exceeded the accuracy of individual predictors [14–16]. Meanwhile, the nomogram compared to the traditional COX analysis presented the exact individual probability of CSS rate but not a conventional notion of relative risk. Moreover, using Harrell's concordance index, AUC, and calibration curve, which were applied to evaluate the performance of the model, was also an advantage over the conventional Cox regression model. Lastly, a risk classification system based on the nomogram's points was constructed for individual patient counselling and follow-up schedule [10].

Simultaneously, we compared our model's clinical value and performance with the AJCC TNM system by the C-index and AUC. As the results show, the nomogram obtained a better C-index (0.733 vs. 0.666) and AUC (1-year, 0.802 vs. 0.697; 3-year, 0.769 vs. 0.7; and 5-year, 0.799 vs. 0.736) compared to the TNM system in the development cohort. Similar results were observed in the vitiation cohort. These results confirmed that our model had better discriminative ability and accuracy for predicting 1-, 3-, and 5-year CSS rates compared to the traditional AJCC classification.

This novel nomogram incorporated seven factors associated with CSS of MIBC patients after PC, including age, histology, T stage, N stage, M stage, regional nodes examined, and tumour size. The T stage was the most significant contributor to the model. Multiple studies indicated that the depth of bladder invasion significantly affected the prognosis of bladder cancer because of its being more aggressive and progressive with the more advanced T stage [17–19]. Meanwhile, PC as an incomplete cancer operation failed to achieve a radical cure if the tumour had regional metastasis such as prostate, uterus, vagina, or abdominal wall [3, 11, 13]. In addition, the nomogram showed that the number of lymph nodes examined had a crucial effect on the CSS of MIBC patients after PC. Lymph node dissection also had an advantage over other bladder preservation strategies like transurethral resection of the bladder (TURB), in that it could simultaneously remove sick lymph nodes and provide pathological information of lymph nodes [20]. Similar to RC, MIBC patients after PC with more lymph nodes removed had a better survival outcome than those without [21, 22]. Interestingly, MIBC patients with adenocarcinoma tended to gain a better CSS than those with TCC when they received PC. However, most previous studies obtained contradictory results [23, 24]. More similar prospective studies were needed to confirm this result. A noteworthy condition was that, in the results of multivariate Cox regression, we did not find statistical differences in survival outcomes between patients with and without radiotherapy and chemotherapy. This might be because we lacked more detailed information about radiotherapy and chemotherapy, like the course of treatment and the type of chemotherapy drugs, which led to our failure to further examine the effects of radiotherapy and chemotherapy in MIBC patients after PC. Meanwhile, the therapeutic effect of radiotherapy still lacked strong evidence to confirm it, and it might need more evidence to verify our results.

To the best of our knowledge, this is the first study to establish a prognostic nomogram for CSS in MIBC patients after PC. However, several limitations should be noted in this study. First of all, our study was a retrospective study based on the SEER database. Thus, the inherent selection biases might undermine the efficacy of our model. Meanwhile, we excluded lots of patients (*n* = 480) whose variable information was unknown, and it was also an essential part of the selection biases. In addition, the SEER database collected cancer information from plenty of different hospitals, and it is difficult to gain a standardized surgical approach. Moreover, the information of carcinoma in situ (CIS), recurrence, and progression was not obtained in the SEER database. Simultaneously, some novel treatments such as robot-assisted surgery, neoadjuvant chemotherapy, and molecular targeted therapy were also vital variables but lacking in the SEER database. Lastly, internal verification was performed in the vitiation cohort, while the patients in the development cohort and vitiation cohort all came from the same database. This verification method is not perfect. Thus, external validation was needed to be performed in a large prospective clinical trial.

## 5. Conclusion

We constructed a highly discriminative and precise nomogram and a corresponding risk classification system which were used to predict the cancer-specific survival for muscle-invasive bladder cancer patients undergoing partial cystectomy. The validation of the nomogram confirmed its excellent performance and applicability. This nomogram can work on the decision on the choice of treatment, patient counselling, and follow-up schedule. Nonetheless, external validation is needed for widely applying.

## Figures and Tables

**Table 1:** Baseline demographical and clinicopathological characteristics of patients.

Characteristics	Total cohort *N* (%)	Development cohort *N* (%)	Validation cohort *N* (%)	*P* value
Number of patients	866	608	258	
Median age (25th–75th percentile)	72 (62–82)	72 (62–82)	72 (62–82)	0.274
Mean age	71.03	71.32	70.35	0.281
*Age*				
<60	155 (17.9%)	99 (16.3%)	56 (21.7%)	0.254
60–70	201 (23.2%)	145 (23.8%)	56 (21.7%)	
70–80	240 (27.7%)	168 (27.6%)	72 (27.9%)	
>80	270 (31.2%)	196 (32.2%)	74 (28.7%)	

*Sex*				
Female	239 (27.6%)	170 (28.0%)	69 (26.7%)	0.714
Male	627 (72.4%)	438 (72.0%)	189 (73.3%)	

*Race*				
White	735 (84.9%)	514 (84.5%)	221 (85.7%)	0.906
Black	73 (8.4%)	52 (8.6%)	21 (8.1%)	
Others	58 (6.7%)	42 (6.9%)	16 (6.2%)	

*Marital status*				
Married	565 (65.2%)	394 (64.8%)	171 (66.3%)	0.730
SDW	213 (24.6%)	149 (24.5%)	64 (24.8%)	
Single	88 (10.2%)	65 (10.7%)	23 (8.9%)	

*Grade*				
Grade I or Grade II	125 (14.4%)	89 (14.6%)	36 (14.0%)	0.793
Grade III or Grade IV	741 (85.6%)	519 (85.4%)	222 (86.0%)	

*Histology*				
TCC	694 (80.1%)	484 (79.6%)	210 (81.4%)	0.610
SCC	60 (6.9%)	41 (6.7%)	19 (7.4%)	
Adenocarcinoma	112 (12.9%)	83 (13.7%)	29 (11.2%)	

*T Stage*				
T2	339 (39.1%)	231 (38.0%)	108 (41.9%)	0.159
T3	458 (52.9%)	322 (53.0%)	136 (52.7%)	
T4	69 (8.0%)	55 (9.0%)	14 (5.4%)	

*N stage*				
N0	785 (90.6%)	550 (90.5%)	235 (91.1%)	0.773
N1 or N2 or N3	81 (9.4%)	58 (9.5%)	23 (8.9%)	

*M Stage*				
M0	835 (96.4%)	587 (96.5%)	248 (96.1%)	0.760
M1	31 (3.6%)	21 (3.5%)	10 (3.9%)	

*Tumor size*				
<3 cm	414 (47.8%)	291 (47.9%)	123 (47.7%)	0.960
>3 cm	452 (52.2%)	317 (52.1%)	135 (52.3%)	

*Number of tumors*				
Single	546 (63.0%)	391 (64.3%)	155 (60.1%)	0.238
Multiple	320 (37.0%)	217 (35.7%)	103 (39.9%)	

*Radiotherapy*				
No/unknown	760 (87.8%)	531 (87.3%)	229 (88.8%)	0.559
Yes	106 (12.2%)	77 (12.7%)	29 (11.2%)	

*Chemotherapy*				
No/unknown	588 (67.9%)	410 (67.4%)	178 (69.0%)	0.653
Yes	278 (32.1%)	198 (32.6%)	80 (31.0%)	

*Regional nodes examined*				
Non	397 (45.8%)	281 (46.2%)	116 (45.0%)	0.830
1–3	133 (15.4%)	95 (15.6%)	38 (14.7%)	
>3	336 (38.8%)	232 (38.2%)	104 (40.3%)	

*Survival time (month)*				
Mean	50.76	48.63	55.78	0.033^※^
Median (25th–75th percentile)	38.5 (13–77.3)	37 (13–73)	41.5 (12.75–92.25)	0.123

TCC: transitional cell carcinoma, SCC: squamous cell carcinoma; other races: American Indian, Alaska Native, Asian, and Pacific Islander; SDW: separated, divorced, or widowed; ^※^: statistical difference.

**Table 2:** Univariate and multivariate regression analyses for CSM.

Characteristics	Univariate analysis		Multivariate analysis	
	HR (95% CI)	*P* value	HR (95% CI)	*P* value
*Age*				
<60	Ref.		Ref.	
60–70	1.311 (0.862–1.994)	0.206	1.143 (0.743–1.759)	0.544
70–80	1.864 (1.249–2.782)	0.002^※^	1.633 (1.075–2.479)	0.021^※^
>80	2.705 (1.841–3.975)	<0.001^※^	2.289 (1.525–3.434)	<0.001^※^

*Sex*				
Female	Ref.			
Male	0.926 (0.719–1.192)	0.551		

*Race*				
White	Ref.			
Black	1.081 (0.731–1.599)	0.697		
Others	0.844 (0.529–1.346)	0.476		

*Marital status*				
Married	Ref.			
SDW	1.267 (0.968–1.657)	0.084		
Single	1.155 (0.804–1.659)	0.436		

*Grade*				
Grade I or Grade II	Ref.			
Grade III or Grade IV	1.891 (1.281–2.793)	0.001^※^		

*Histology*				
TCC	Ref.		Ref.	
SCC	1.787 (1.183–2.699)	0.006^※^	2.447 (1.537–3.894)	<0.001^※^
Adenocarcinoma	0.440 (0.287–0.675)	<0.001^※^	0.818 (0.468–1.428)	0.479

*T Stage*				
T2	Ref.		Ref.	
T3	3.102 (2.309–4.168)	<0.001^※^	3.395 (2.508–4.597)	<0.001^※^
T4	5.288 (3.586–7.798)	<0.001^※^	3.956 (2.668–5.866)	<0.001^※^

N stage			2.668–5.866	
N0	Ref.		Ref.	
N1 or N2 or N3	2.019 (1.449–2.813)	<0.001^※^	2.277 (1.568–3.308)	<0.001^※^

*M Stage*				
M0	Ref.		Ref.	
M1	6.426 (4.030–10.247)	<0.001^※^	4.872 (2.965–8.007)	<0.001^※^

*Tumor size*				
<3 cm	Ref.		Ref.	
>3 cm	1.475 (1.169–1.861)	0.001^※^	1.334 (1.055–1.688)	0.016^※^

*Number of tumor*				
Single	Ref.			
Multiple	0.961 (0.756–1.223)	0.749		

*Radiotherapy*				
No/unknown	Ref.		Ref.	
Yes	1.379 (1.003–1.895)	0.048^※^	1.059 (0.761–1.473)	0.734

*Chemotherapy*				
No/unknown	Ref.			
Yes	0.908 (0.710–1.162)	0.444		

*Regional nodes examined*				
Non	Ref.		Ref.	
1–3	0.678 (0.522–0.879)	0.003^※^	0.568 (0.396–0.815)	0.002^※^
>3	0.745 (0.540–1.029)	0.074	0.538 (0.406–0.713)	<0.001^※^

TCC: transitional cell carcinoma, SCC: squamous cell carcinoma; other races: American Indian, Alaska Native, Asian, and Pacific Islander; SDW: separated, divorced, or widowed; ^※^: statistical difference

**Table 3:** Nomogram scoring system.

Variables	Points	Variables	Points	Variables	Points
Age		M stage		Regional nodes examined	
<60	66.13	M0	89.64	Non	0
60–70	59.53	M1	0	1–3	37.77
70–80	26.86	Tumor size		>3	42.05
>80	0	<3 cm		T stage	
Histology		>3 cm		T2	100
TCC	46.34	N stage		T3	17.60
SCC	0	N0	52.62	T4	0
Adenocarcinoma	82.92	N1 or N2 or N3	0		
1-year CSS probability		3-year CSS probability		5-year CSS probability	
0.1	70	0.1	150	0.1	171
0.2	117	0.2	191	0.2	218
0.3	142	0.3	215	0.3	243
0.4	170	0.4	241	0.4	263
0.5	192	0.5	257	0.5	283
0.6	219	0.6	282	0.6	311
0.7	238	0.7	302	0.7	331
0.8	270	0.8	331	0.8	
0.9	311	0.9		0.9	

## Data Availability

The data in this article are obtained from the SEER database (https://seer.cancer.gov/data/). The data from SEER are publicly available and deidentified.
